# Transient Thermomechanical Simulation of 7075 Aluminum Contraction around a SiO_2_ Microparticle

**DOI:** 10.3390/ma14010134

**Published:** 2020-12-30

**Authors:** Pedro Alejandro Tamayo-Meza, Miguel Ángel Cerro-Ramírez, Emmanuel Alejandro Merchán-Cruz, Usiel Sandino Silva-Rivera, Raúl Rivera-Blas, Luis Armando Flores-Herrera

**Affiliations:** 1 Postgraduate Studies and Research Section, Instituto Politecnico Nacional, Higher School of Mechanical and Electrical Engineering, U. Azcapotzalco, Av. Granjas 682, Mexico City 02250, Mexico; eamerchan@ipn.mx (E.A.M.-C.); rriverab@ipn.mx (R.R.-B.); 2 Mechatronics Engineering Department, Tecnológico de Estudios Superiores de Coacalco, Av. 16 de Septiembre 54, Coacalco de Berriozábal 55700, Edo. de Mex., Mexico; miguel_cerro.ccai@tesco.edu.mx; 3 SEDENA, D.G.E.M., Escuela Militar de Ingenieros, Secc. of Industrial Engineering, Army and Air Force University, Escuela Militar de Ingenieros, Av. Industria Militar 261, Naucalpan de Juarez 53960, Edo. de Mex., Mexico; ussilvar@ing-mil.com

**Keywords:** microparticle, stress state, plate defects, simultaneous cooling

## Abstract

One important challenge that faces the metallurgic industry turns around the constant increment in the mechanical resistance of certain finished products. Metallurgic advantages can be obtained from the inclusion of microparticles in metallic materials, but this inclusion involves complex challenges as the internal stress distribution can be modified. In this work, the simulation of a cooling sequence in 7075 aluminum with a SiO_2_ microparticle is presented. Two models of two-dimensional (2D) type were constructed in ANSYS^®^2019 with circular and oval shape microparticles located inside the aluminum. Both models were subjected to the same thermomechanical transient analysis to compare the remaining stress distributions around the microparticles after the thermal load and to observe the effect of the geometrical shape. The results show remaining stresses increased in the oval model as a consequence of the geometrical shape modification. After applying a tension load in the analyzed specimens, shear stress concentrations were observed with a higher magnitude around the covertex of the oval shape. The results can be very useful for the creation of materials with controlled remnant stress located in specific or desired locations in the matrix.

## 1. Introduction

The size, shape, composition, and concentration of the non-metallic inclusions play a fundamental role in one of the main properties of the alloy, in this case, the viscosity. The presence of defects in metallic matrices can create cracks that propagate depending on their size, geometry, and orientation [1,2]. On the other hand, the presence of inclusions and microparticles creates stress concentration points that modify the mechanical behavior of the material, its resistance, and its machinability [3,4,5,6], especially when they are subjected to considerable temperature treatments [7,8,9].

Liquid metal foundries have a micro heterogeneous structure, in which three structural zones can be distinguished: (a) a zone with relatively stable solid formations, with a useful life of τ: 10^−9^ to 10^−10^ s and dimensions of 10–100 Å; (b) a zone of active atoms, which are simultaneously part of the groups (clusters) and have greater energy and mobility; and finally, (c) a zone of free space volume, which includes the region of bond breaking between the groups and appears and disappears constantly in the process of thermal vibration of the clusters (groups).

Oxides, sulfides, pores, silicates, and nitrogen, in the form of non-metallic inclusions, sensitively affect the mechanical resistance of metals [10,11,12,13,14,15]. The non-metallic inclusions are potential stress concentrators and the source of crack incubation processes that can lead to a desired mechanical behavior if they are properly controlled. Oxide particles, such as Al_2_MgO_4_, Al_2_O_3_, MgO, and SiO_2_, observed in aluminum alloys are extremely hard concerning the matrix and their treatment represent a complex technological challenge [16,17,18,19,20,21,22]. The addition of microparticles in metals, combined with proper thermomechanical treatment, can be used to increase the mechanical properties in a beneficial sense [23,24,25,26,27,28,29,30,31,32].

## 2. Materials and Methods

### 2.1. Problem Description

The study was carried out with consideration of the main characteristics of Al 7075 (Al-Zn-Mg-Cu type), together with Al 2024 (Al-Cu-Mg type). The pressing recrystallization temperature ranges between 300 and 350 °C and the non-pressing recrystallization temperature is between 420 and 450 °C. The structural alteration of aluminum alloys is related to the modification of their chemical properties. In this phenomenon, the greatest influence is exerted by second phase intermetallic inclusions, which are precipitated from the melt and distributed along both the grain edges and the inter-dendritic zones. These particles can modify the general characteristics of the alloy as well as its resistance to fracture. Second phase inclusions, such as Al_2_O_3_, CuAl_2_, and others, create microcracks, the irregularly shaped particles that break near the matrix interface. Microcracks arising from the inclusions are transferred to the slip bands, which generate thin regions along the trajectory. By increasing the volumetric portion of these second phases, the relative elongation is reduced by approximately 5.1% to 2.9%, that is, by 1.8 times. This effect is catastrophic when manufacturing mechanical implements, mechanical structures, or mechanisms in engineering [33,34,35,36].

The chemical composition of the alloy is a fundamental issue in the formation of the structure and the intermetallic inclusions. It is, therefore, very timely to now share the following case that we have developed for an alloy of the Al-Zn-Mg-Cu system [37]. This alloy was composed of 5.7% of Zn, 2.2–2.7% of Mg, 1.5–2.0% of Cu, 0.11–0.17% of Zr, and an amount of Fe and Si < 0.15%. From this combination, we obtained a *σ*_0.2_ = 49.5 kg/mm^2^, *K_1c_* = 123 kg/mm^3/2^, and *K_1c_* = 95 kg/mm^3/2^. Subsequently, we developed the following set of modifications in the chemical composition: 5.6% of Zn, 2.5% of Mg, 1.6 of Cu, 0.7% of Fe, 0.5 of Si, and 0.2% of Cr. From this combination, we obtained a *σ*_0.2_ = 40 kg/mm^2^, *K_1c_* = 88 kg/mm^3/2^, and *K_1c_* = 76 kg/mm^3/2^. From these results, we deduced that the alloy in the Al-Zn-Mg-Cu system with a lower content of Fe and Si, and that contained Zr instead of Cr, presented better toughness and resistance to crack propagation caused during fatigue loads and higher resistance to corrosion with respect to the widely known 7075-T765.

In recent years, some authors have expressed concerns about the generality of the principle of orientation for boundary conditions. At the same time, electrochemical theories have made it possible to consider the influence of important factors, such as the electrical nature of the metal and the inclusion. The idea of an electrochemical approach towards the interaction of refractory, insoluble inclusions with liquid metal is based on a change in the level of the Fermi energy of two substances in close contact and the formation of an electrical double layer at the contact surface [38].

### 2.2. Construction of the Geometry and Boundary Conditions

**Figure 1:**
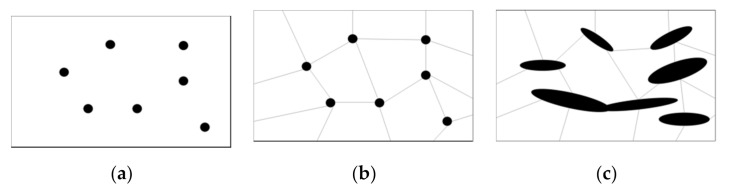
(**a**) Random distribution of the microparticles in the matrix, (**b**) location of the microparticles in boundary grains intersections, and (**c**) variability of microparticle size and shape.

Figure 1a shows a random distribution of microparticles (dots) within the matrix. This could be an idealized condition used to calculate a stress distribution across the material. However, the number of variables that can be presented in the real physical scenario is more complex. For example, as shown in Figure 1b, the microparticles could be located at the intersections of grains, or, as shown in Figure 1c, variable sizes and shapes of microparticles could create shorter or larger distances between each other, thus modifying the wear resistance or some other mechanical requirement [31,32]. Up to this point in our research, we have focused only on the analysis of a single microparticle and its stress distribution when subjected to a contraction process caused by changes in temperature. Microparticles can have different geometrical shapes and sizes; additionally, they can be distributed in the metallic matrix without a specific order.

To simplify a single scenario of this problem, we consider a circular microparticle with radius *r* located inside a metallic matrix as shown in Table 1. Consider now, from an initial temperature, that both materials are in a cooling sequence. Assume that the matrix achieves an initial temperature *T_i_*, reduced up to *T_f_*, which can be room temperature for this example. Based on this concept, only two-dimensional (2D) models were constructed. They have a squared shape, are 1 mm in length, and are oriented in the *x,y* plane. The second and third columns indicate the location of the loads for the Transient Thermal and Static analyses. Inside the square, a proposed circular shape microparticle with a 0.1 mm diameter was located for the first case. The circular microparticle has an equivalent area of 0.0314 mm^2^. In the second case, the oval microparticle was extended in the *x*-axis with a length of 0.2 mm and an equivalent area of 0.094 mm^2^.

### 2.3. Numerical Model Validation

**Figure 2:**
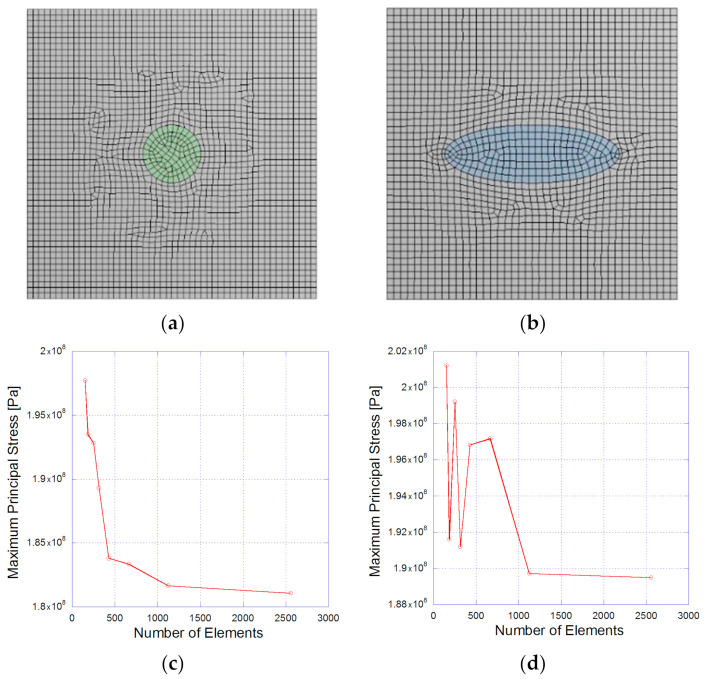
A resulting mesh of the aluminum matrix with (**a**) the circular microparticle, (**b**) the oval microparticle, the maximum principal stress for the number of elements for (**c**) the circular microparticle, and (**d**) the oval microparticle.

Figure 2 shows the resulting continuous mesh type of the numerical models obtained with the meshing module of the ANSYS^®^ software (2019-R1-Academic, Ansys Inc., Canonsburg, PA, USA): Figure 2a for the circular microparticle and Figure 2b for the oval microparticle. A series of previous static analyses were carried out to validate both numerical models; these were used to observe the resulting mesh dependence. Figure 2c,d show the relationship between the stress levels for the circle and the oval. In both cases, the results stabilized after increasing the mesh by 1000 elements. The final mesh for the circular model consisted of 2500 elements with an average skewness value of 0.03768 and average orthogonality of 0.994. For the oval model, the resulting mesh was finally composed of 1855 elements with an average skewness value of 0.0527 and average orthogonality of 0.9923. These values correspond to an excellent mesh quality level.

### 2.4. Material Properties

The purpose of the present simulation was to observe the stress increment during the contraction of the 7075 aluminum [39,40,41], and according to a predefined cooling sequence in which the thermal and mechanical properties change. These properties are shown in Table 2 and Table 3. Table 4 shows the thermal and mechanical properties considered for the SiO_2_ inclusion [42,43]. These values were proposed and implemented in the simulation as input parameters during the pre-processing set up for the thermal and mechanical fields.

### 2.5. Transient Thermal and Static Analysis

**Figure 3:**
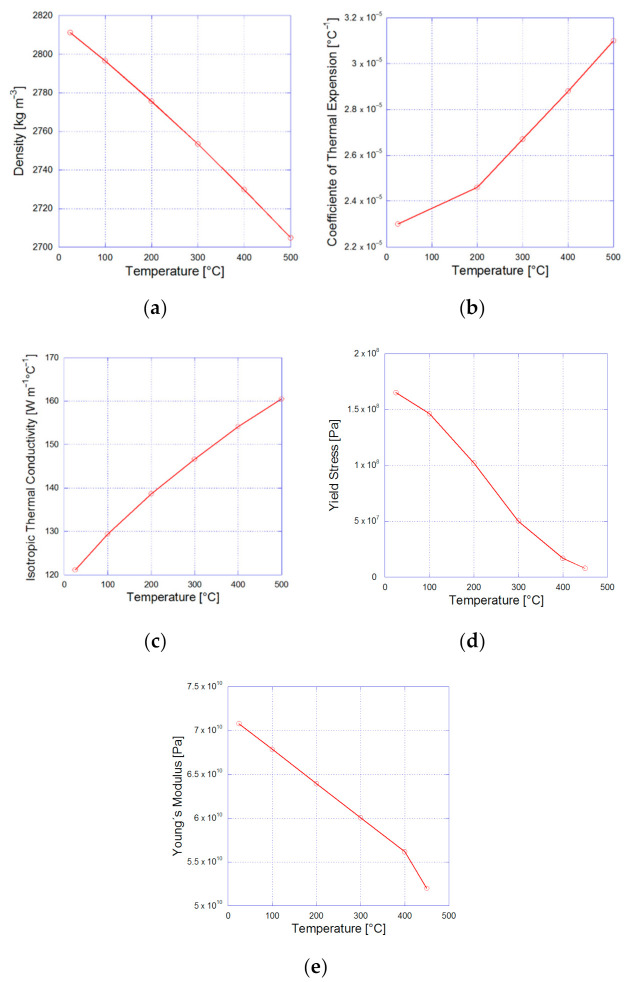
Variations are considered for every temperature modification with respect to (**a**) the density, (**b**) the coefficient of thermal expansion, (**c**) the isotropic thermal conductivity, (**d**) the yield stress, and (**e**) Young’s modulus.

The analysis considers the interaction between two physical fields carried out with the Transient Thermal and Static Structural modules of the ANSYS^®^ 2019R1 software [44]. The analysis is divided into 50 step times; the thermal field is solved first, followed by the mechanical field. The thermal load was applied from step numbers 0 to 29 and the static load from step numbers 30 to 50. The initial conditions of the thermal analysis considered the temperature applied on the external faces of the 2D model of 1 mm by 1 mm in length and height; the radius of the microparticle is 0.1 mm. The initial temperature applied on the thermal analysis started at 400 °C and it was reduced every step time to 25 °C. The static analysis started at step number 30 by applying a tension load that increased linearly from 0 to 0.16 N with increments of 0.001 N. The matrix basement was constrained in the form of a frictionless surface on the horizontal *x*-axis. Figure 3 shows the physical properties of the 7075 aluminum with respect to temperature considered for the simulation; the density is shown in Figure 3a, the coefficient of thermal expansion in Figure 3b, the isotropic thermal conductivity in Figure 3c, the yield stress in Figure 3d, and, finally, Figure 3e shows the modification in Young’s modulus.

## 3. Results

**Figure 4:**
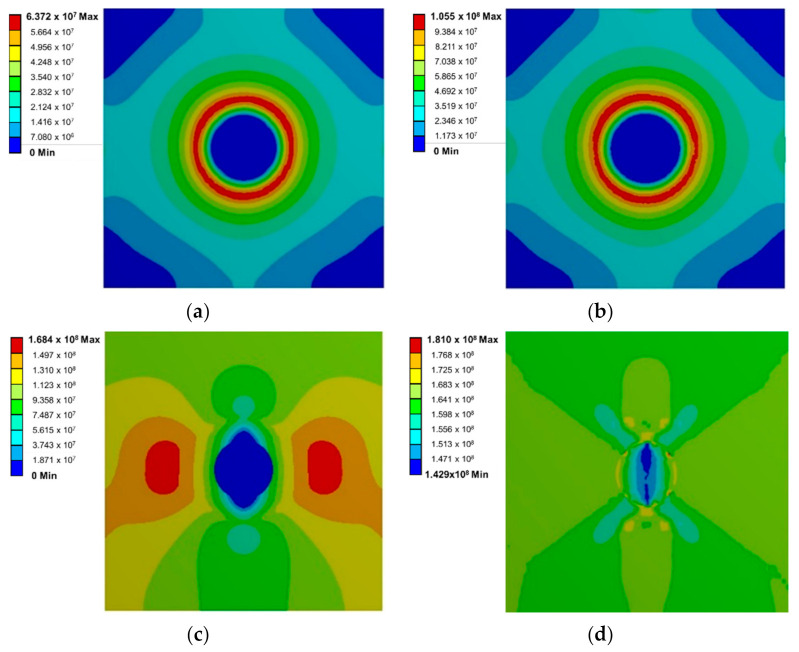
Stress distribution around the circular SiO_2_ microparticle for (**a**) step number 10, (**b**) step number 20, (**c**) step number 40, and (**d**) step number 50.

Figure 4 shows the mechanical behavior of the sphere subjected to the Transient Thermal load around the circular SiO_2_ microparticle. In Figure 4a, the distribution of stresses for step number 10 is observed, and in Figure 4b, step number 20 shows a similar distribution, but with increased magnitude. During the contraction of the material, the microparticle presents a state of compressive stresses. From the boundary, a state of tensile stresses distributed around the microparticle arises. Subsequently, the normal tension load generates the appearance of shear stress planes as shown in Figure 4c, which corresponds to step number 40, and in Figure 4d, which corresponds to step number 50. The maximum shear stress planes are observed at approximately 45°. It can be observed that the maximum stress is located at the beginning of the cutting plane.

**Figure 5:**
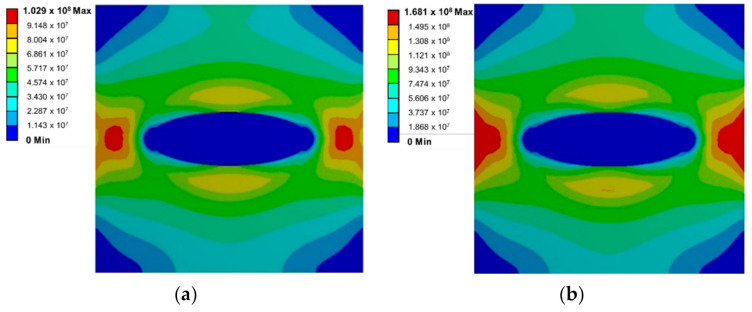

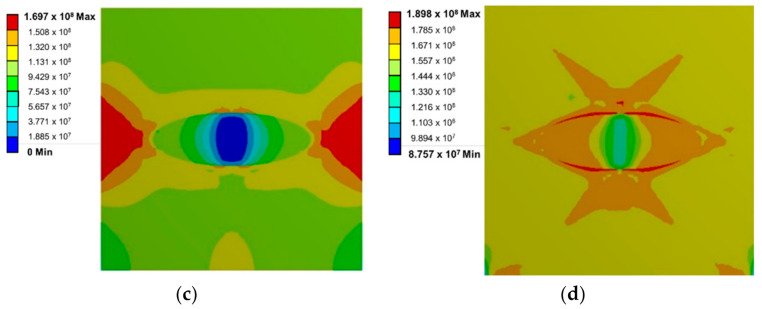
Stress distribution around the oval SiO_2_ microparticle for (**a**) step number 10, (**b**) step number 20, (**c**) step number 40, and (**d**) step number 50.

Figure 5 shows the mechanical behavior of the oval SiO_2_ microparticle subjected to the Transient Thermal load. Figure 5a shows the stress distribution for step number 10, and Figure 5b for step number 20. Unlike the case of the circular microparticle during the contraction of the material, the stress is concentrated in the vertex of the oval microparticle. Subsequently, it is subjected to a normal tension load and the appearance of shear stress planes are shown in Figure 5c, which corresponds to step number 40, and in Figure 5d, which corresponds to step number 50. The maximum stress is finally concentrated along the surface of the covertex.

**Figure 6:**
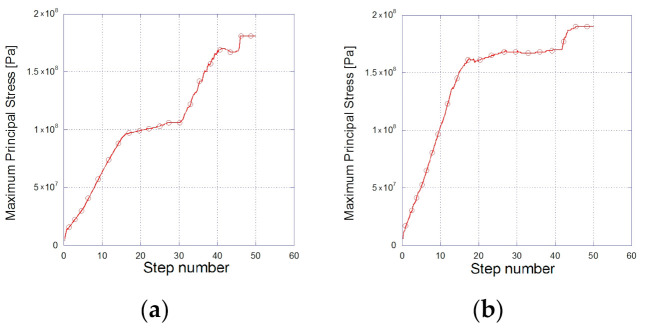
Maximum principal stress during the transient thermomechanical analysis for (**a**) the circular and (**b**) the oval microparticle.

Figure 6 shows the maximum principal stress with respect to the step number, in Figure 6a for the circular and Figure 6b for the oval microparticle.

**Figure 7:**
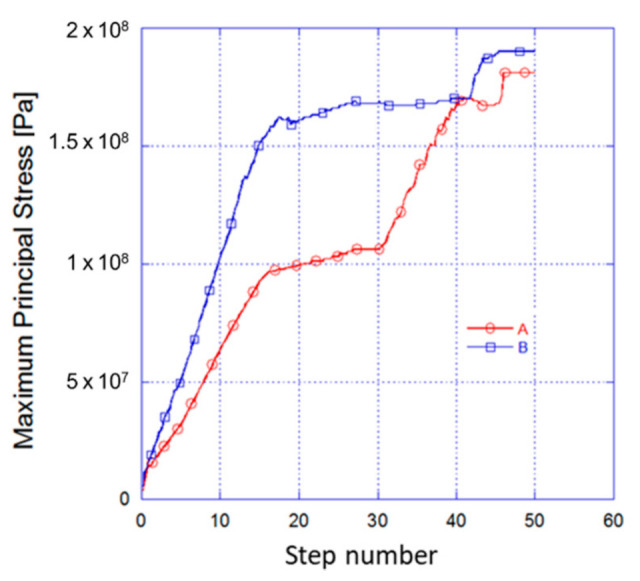
Stress magnitude comparison between A the circular and B the oval microparticle.

Figure 7 shows a comparison of stress between both cases with A for the circular and B for the oval microparticle. The oval microsphere has a more adverse effect on the elastic zone of the material as shown in line B. The oval microsphere increases the ultimate stress after having exceeded the yield stress, which was modified during the Transient Thermal process. The oval geometry increases the maximum stress by 169 MPa for step number 27. In the same step, the circular microparticle reaches a value of 106 MPa. This is the point of greatest difference with a total of 63 MPa. It can be observed that as the irregularity of the shape of the microparticle increases, the increment of local stress increases, indicating possible failure appearances.

## 4. Discussion

The presence of microparticles immersed in metallic materials can be observed from a dangerous point of view if they are added to the presence of microcracks, inclusions, dislocations, and other types of uncontrolled defects. However, in another sense, they can be beneficial to increase mechanical capabilities. A controlled inclusion of microparticles can be applied to achieve surface improvements under wear conditions using non-metallic materials, as in the case presented here, in which an oxide has been included. The inclusion of this aggregate requires considering a uniformly controlled distribution of the microparticles to achieve the desired distribution of the remaining stresses in their magnitude, direction, and distribution. The proposed model is not a straight postulate, but it is the result of previous analysis related to changes observed during fusion processes. The fundamental characteristic of this model and the method for the analysis is extensive and non-additive, as in the case of analyzing some structurally sensitive properties of metals in the liquid state and their interaction with intermetallic particles arising within the liquid. This means that the entire mass of liquid is part of the phenomena developed in the system.

## 5. Conclusions

The results obtained from the simulation revealed that during the contraction sequence greater compression stress was generated around the microparticle, as expected; but, it is important to notice that the model of the microparticle, in this case, considered idealized conditions. The real shape, size, and distribution of the microparticles must deal with the interaction of grain dynamics, physical characteristics, and dislocations that can arise during thermal treatments. The most important observation found with this simulation was the appearance of non-linearly distributed shear stresses with a higher magnitude around the covertex of the oval shape model. These results must be combined with more complex physical scenarios, in which the proximity of other microparticles can be used to deliberately address the magnitude and direction of remaining stresses and thereby obtain a modified or improved mechanical condition. However, this study allows us to observe how the geometrical irregularities of the microparticles tend to increase the appearance and location of stress concentrations caused by thermal or static loads.

## Figures and Tables

**Table 1:** Geometries considered for the Transient Thermal and Static analysis.

Microparticle Orientation	Thermal Load	Mechanical Load
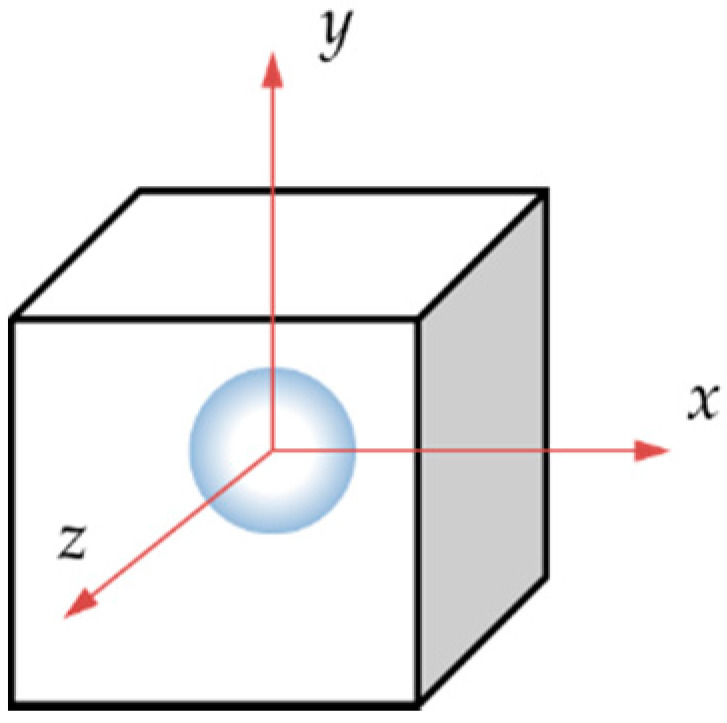	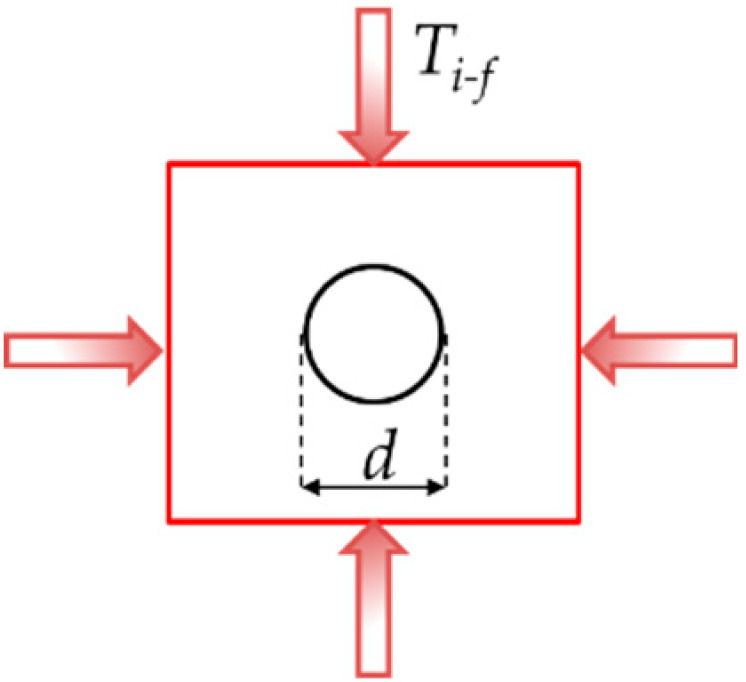	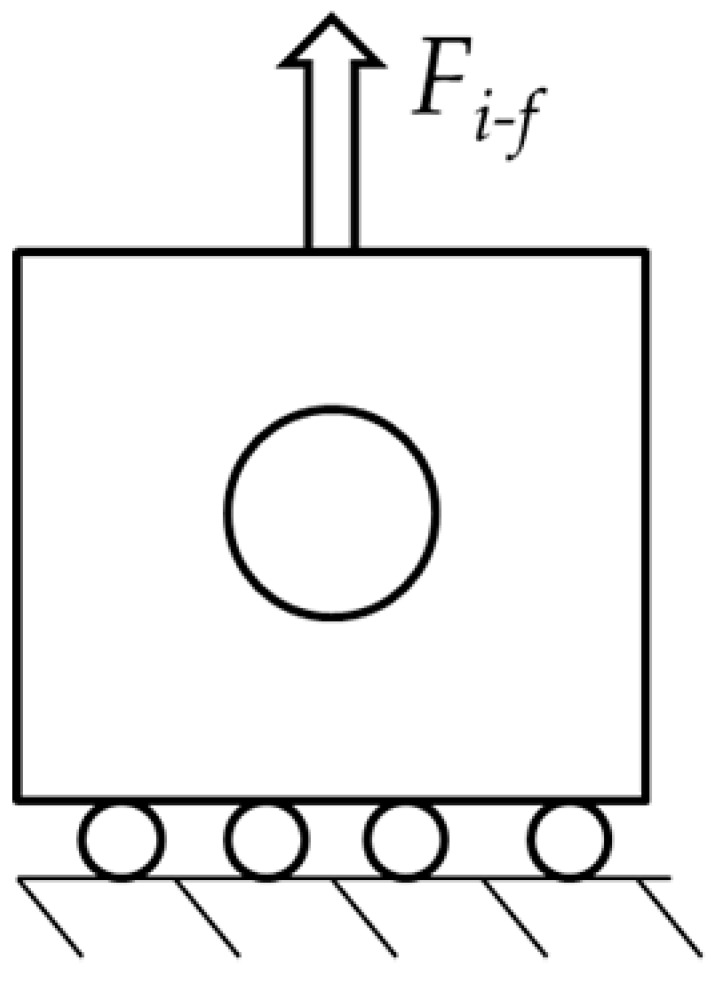
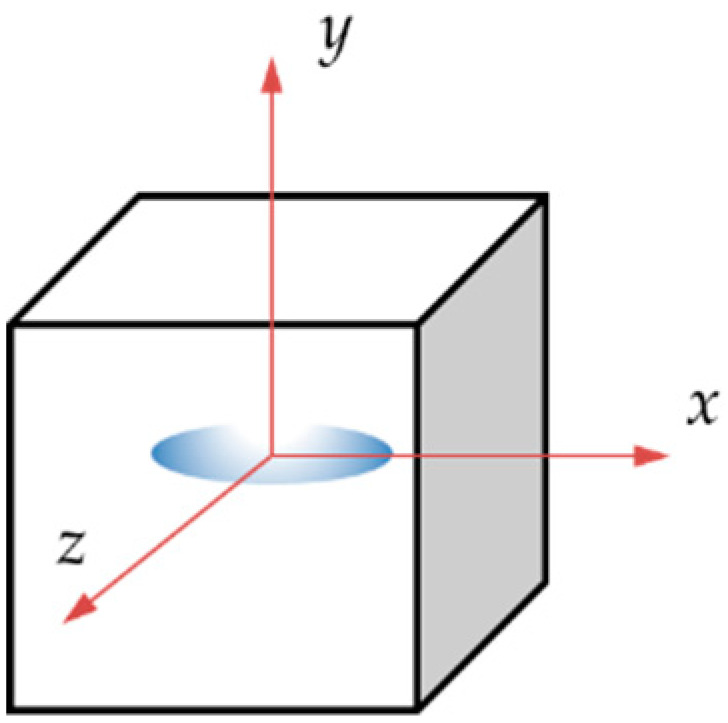	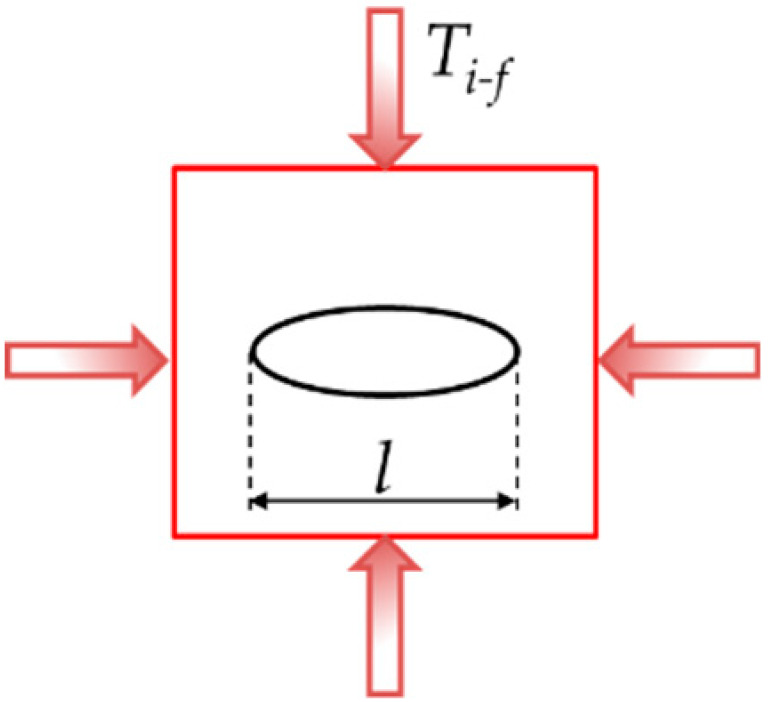	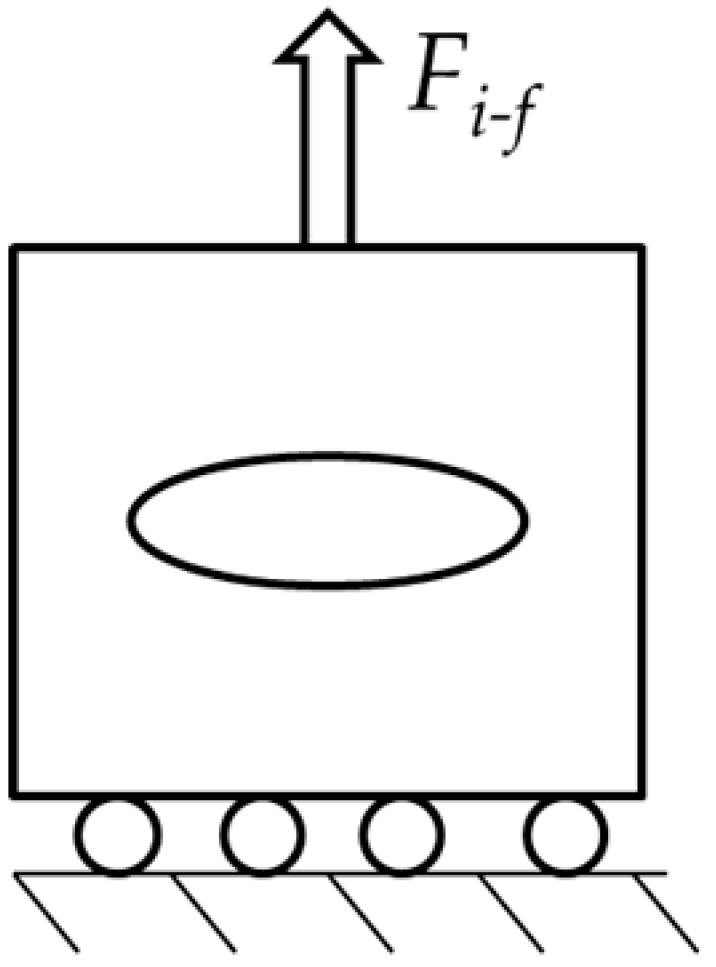

**Table 2:** Considered thermal properties of 7075 aluminum with respect to temperature.

Temperature (°C)	Density (kg·m^−3^)	Coefficient of Linear Expansion (C^−1^)	Thermal Conductivity (Wm^−1^·C^−1^)	Specific Heat (Jkg^−1^·C^−1^)
25	2811.2	2.30 × 10^−5^	121.1	860.4
100	2796.5	2.46 × 10^−5^	129.4	900.7
200	2775.6	2.67 × 10^−5^	138.6	943.7
300	2753.4	2.88 × 10^−5^	146.6	983.6
400	2729.8	3.10 × 10^−5^	154.1	1024.2
500	2704.9	3.33 × 10^−5^	160.5	1136.6

**Table 3:** Considered mechanical properties of 7075 aluminum with respect to temperature.

Temperature (°C)	Young Modulus (GPa)	Poisson Ratio	Yield Stress (MPa)
25	70.7	0.33	165.06
100	67.8	0.33	146.13
200	63.9	0.33	102.08
300	60.0	0.33	50.23
400	56.1	0.33	16.91
450	52.0	0.33	8.03

**Table 4:** Thermal and mechanical properties considered for the SiO_2_.

Thermal Properties	Value
Thermal conductivity (Wm^−1^ C^−1^)	1.5
Specific heat (Jkg^−1^ C^−1^)	745
**Mechanical properties**	
Young modulus (GPa)	66.3
Poisson ratio	0.15
**Initial conditions–Heat transfer by convection**	
Initial temperature (°C)	400
Co-efficient of the transfer layer (Wm^−2^ C^−1^)	15
Environmental temperature (°C)	25

## Data Availability

Data sharing not applicable this article.
